# UAS-based real-time water quality monitoring, sampling, and visualization platform (UASWQP)

**DOI:** 10.1016/j.ohx.2022.e00277

**Published:** 2022-02-11

**Authors:** Jae Hyeon Ryu

**Affiliations:** University of Idaho, United States

**Keywords:** *UASWQP*, Unmanned aircraft system (UAS), Unmanned aircraft vehicle (UAV), Water quality, Internet of Things (IoT), ThingSpeak, Drone

## Abstract

Urbanization, land use change, and agricultural activities continue to affect water quality standards at the urban–rural interface, such as the Boise River System located in Idaho, USA. This project demonstrates how the off-the-shelf unmanned aircraft system (UAS, also known as drone) equipped with other necessary hardware attachments can be used to monitor real-time water quality components, including pH, water temperature, electric conductivity (EC), and dissolved oxygen at open waterbodies. The proposed UAS-based hardware platform for water quality studies (UASWQP) appears a promising tool to advance environmental research activities, especially for impaired waterways (e.g., rivers, lakes, and reservoirs). The preliminary result shows that the proposed UASWQP effectively displays water quality components in real-time to the ThingSpeak Cloud web services, while an adequate water sample was also collected easily for further analysis at laboratory facilities, when needed. It is anticipated that UASWQP will be a useful tool to promote environmental stewardship by contributing to the water research communities in years to come.

## Graphical abstract

**Figure:**
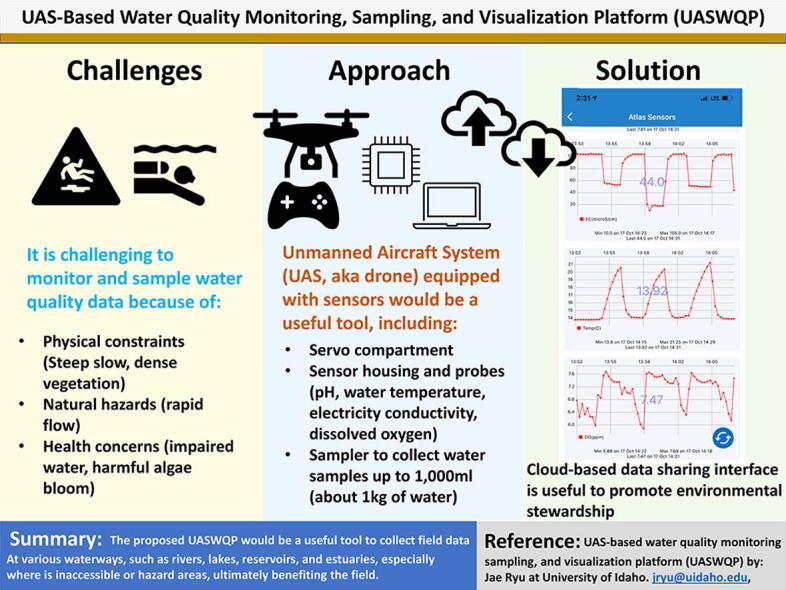

## Hardware in context

As cities undergo significant urban development and population growth, water management and its usage have become more essential. Additionally, agricultural activities near the urban–rural interface, such as the Boise area located in the state of Idaho, USA often contribute to water quality standards at local waterways (e.g., lakes, rivers, reservoirs). The potential risks driven by this circumstance, can be mitigated through extensive and enhanced water monitoring and management exercises. Typical water quality analysis requires time-consuming and labor-intensive field work to bring water samples to laboratory facilities for further analysis. In addition to physical constraints, field crews often face unprecedent challenges associated potential hazardous pollutants, such as harmful algae bloom (HAB), resulting in secondary risks to those parties involved. Autonomous sampling and monitoring using an Unmanned Aircraft System (UAS), therefore, can be an alternative solution to eliminate potential risks.

Thanks to recent technology improvement, water quality monitoring tools are evolving over time. Satellite-based remote sensing technology, wireless sensor network (WSN), and automated monitoring stations (AMS) are commonly used as a supplement to the traditional measurement methods of water collection and subsequent laboratory analysis [1]. For example, Gholizadeh et al. (2016) indicate that satellite-based hyperspectral imageries would be a good approach to detect water quality parameters, such as chlorophyll-a (chl-a) over large areas while the traditional method is still required to make an environmental decisions on policy making [1]. Wireless Sensor Networks (WSNs) is another avenue on water quality monitoring exercises in the sense that it can be in situ and continuous real-time water quality data (e.g., water temperature, pH), and transmit it through a wireless network [2]. The installation and maintenance cost of the WSNs and AMS, however, would be expensive due to their complexity and nature of sensor technology. Besides, spatial, and temporal variability of the data from these sensor networks would be insufficient because of their sparse distribution in a large waterbody [3].

Autonomous underwater vehicles (AUSs) and autonomous surface vehicles (ASVs) have been also used to monitor water quality components [4], [5], [6]. But accurate Global Positioning System (GPS) reception is still challenging for AUS’s operation as a surfacing is required when the distance traveled by the underwater vehicles reaches a certain range [5]. The autonomous surface vehicles (ASVs) are also deployed to monitor water quality although it is difficult to operate wind-driven swaying from side to side and/or uncertain engine-control frequencies [7].

More recently, an UAS-based water quality monitoring platform has been introduced [3], [8]. They proposed a method to measure water quality components, including temperature, electrical conductivity (EC), dissolved oxygen (DO), and pH of water using a custom-built hexacopter. An open-source multiprobe meter (OSMM) was attached to the UAS along with a custom-designed 3D-printed parts and accessories. The result indicates that UAS-based water monitoring and sampling is promising not only because it can be useful to conduct field measurements at inaccessible or dangerous waterbodies but also it is very handy for rapid water quality measurements after natural disasters such as flooding and hurricane events [3].

Although the previous studies contribute to water quality measurements and sampling activities, additional investigation is still needed to enhance real-time water quality monitoring and sampling activities by promoting environmental stewardships. In this study, therefore, the author investigates the feasibility of real-time water quality monitoring and sampling using an off-the-shelf unmanned aircraft, such as DJI Matrice 600 Pro (M600) [9], the largest hexacopter drone available at the off-the-shelf market. The preliminary result indicates that the proposed UAS-based real-time water quality monitoring and sampling platform (UASWQP) could be a promising tool to drive a momentum toward enhancing environmental stewardships, ultimately contributing to water research communities and the public safety.

## Hardware description.

A professional-grade flying platform, such as DJI Matrice 600 (M600) was used as a basic frame to develop real-time water quality monitoring, sampling, and data visualization platform (UASWQP). The M600 can support a total takeoff weight of 15 kg designed for industry applications. The built-in A3 pro flight controller (A3) and three sets of global navigation satellite system (GNSS) units enable precise control of multi-rotor aircraft (hexacopter) by providing accurate data for stable flight performance [9]. The flight time is about 16 min with a maximum payload in range of 5.5–6 kg, including the attached apparatus and a 1000 ml (about 1 kg) sample of water.

**Fig. 1:**
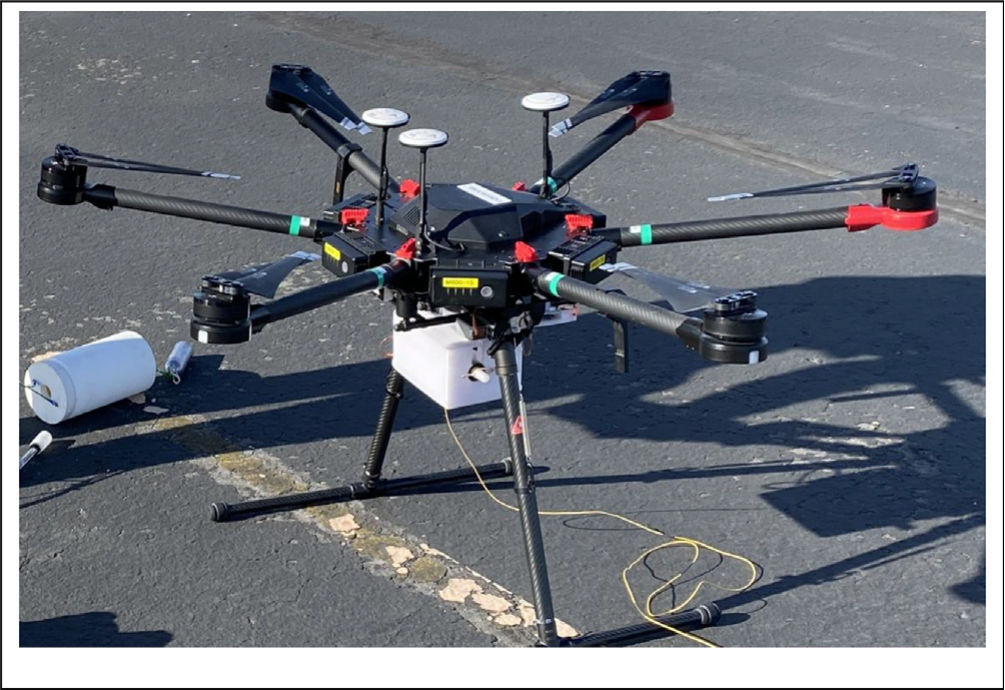
The UAS-based real-time water quality monitoring, sampling, and visualization platform (UASWQP).

**Fig. 2:**
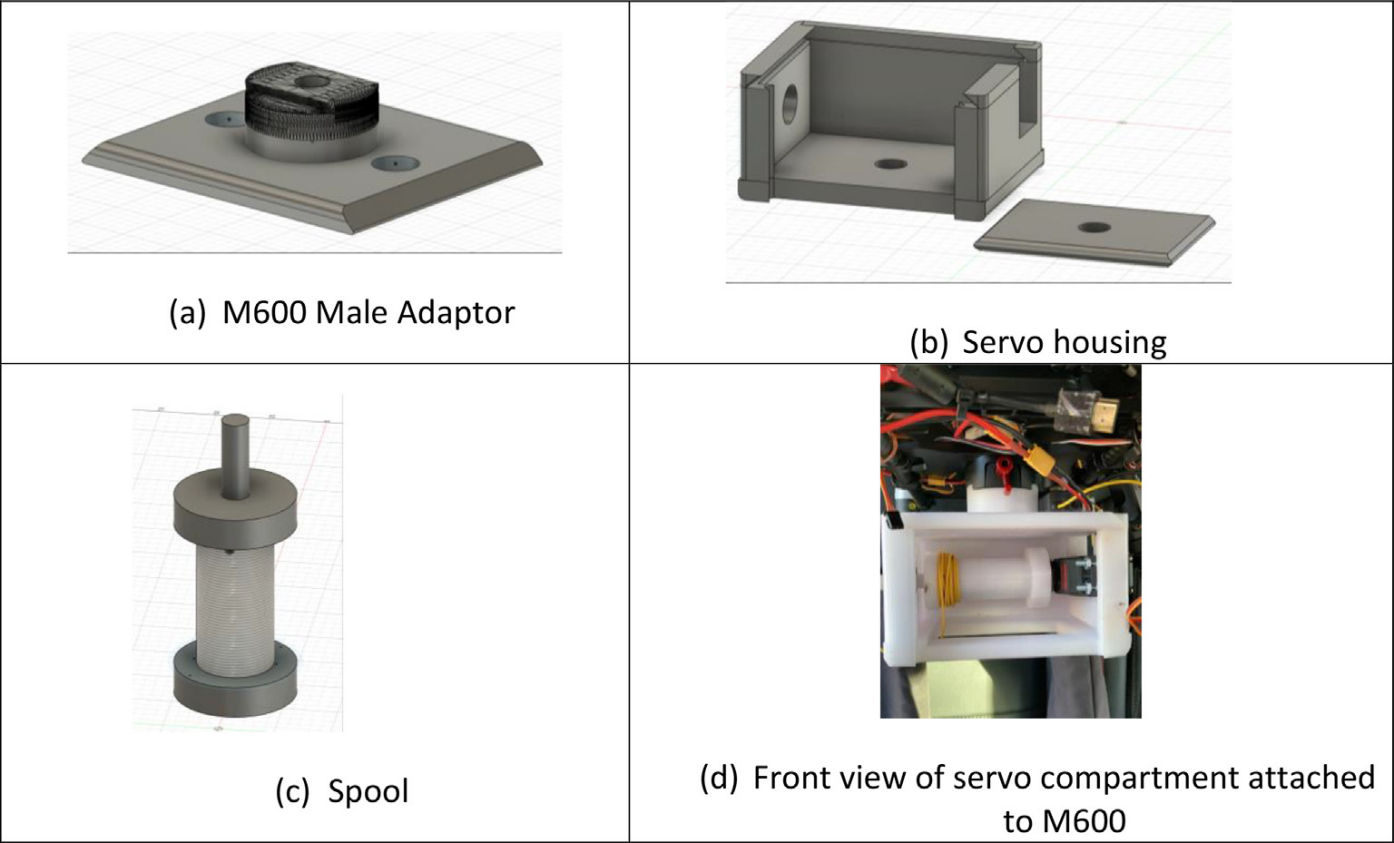
Parts of the servo compartment. (a) M600 Male Adaptor (b) Servo housing, (c) Spool, (d) Front view of servo compartment attached to M600.

The water quality monitoring and sampling apparatus was designed and constructed using 3D-printed parts and then those are attached to underneath M600 as shown in Fig. 1. The apparatus consists of three components: (1) servo compartment, (2) sensor housing and probes, and (3) water sampler and connected with a strong synthetic fiber, such as Kevlar taglines. The servo compartment was first designed and constructed using 3D printed parts that are easily attachable to M600 (See Fig. 1). Note that all parts here are 3D-printed using Fusion 360 software [10]. To increase maximum retraction force, a 60 kg digital servo supporting up to 8.4 voltage was used after minor adjustment for continuous rotations. The power source for the servo was withdrawn from M600 directly via the XT30 plug connector and battery eliminator circuit (BEC) to regulate the voltage to the servo. A necessary effort on electrical wiring, soldering, and port mapping was also needed to connect the built-in A3 flight controller and the servo by which is operational remotely in the ground control station (see Fig. 2).

**Fig. 3:**
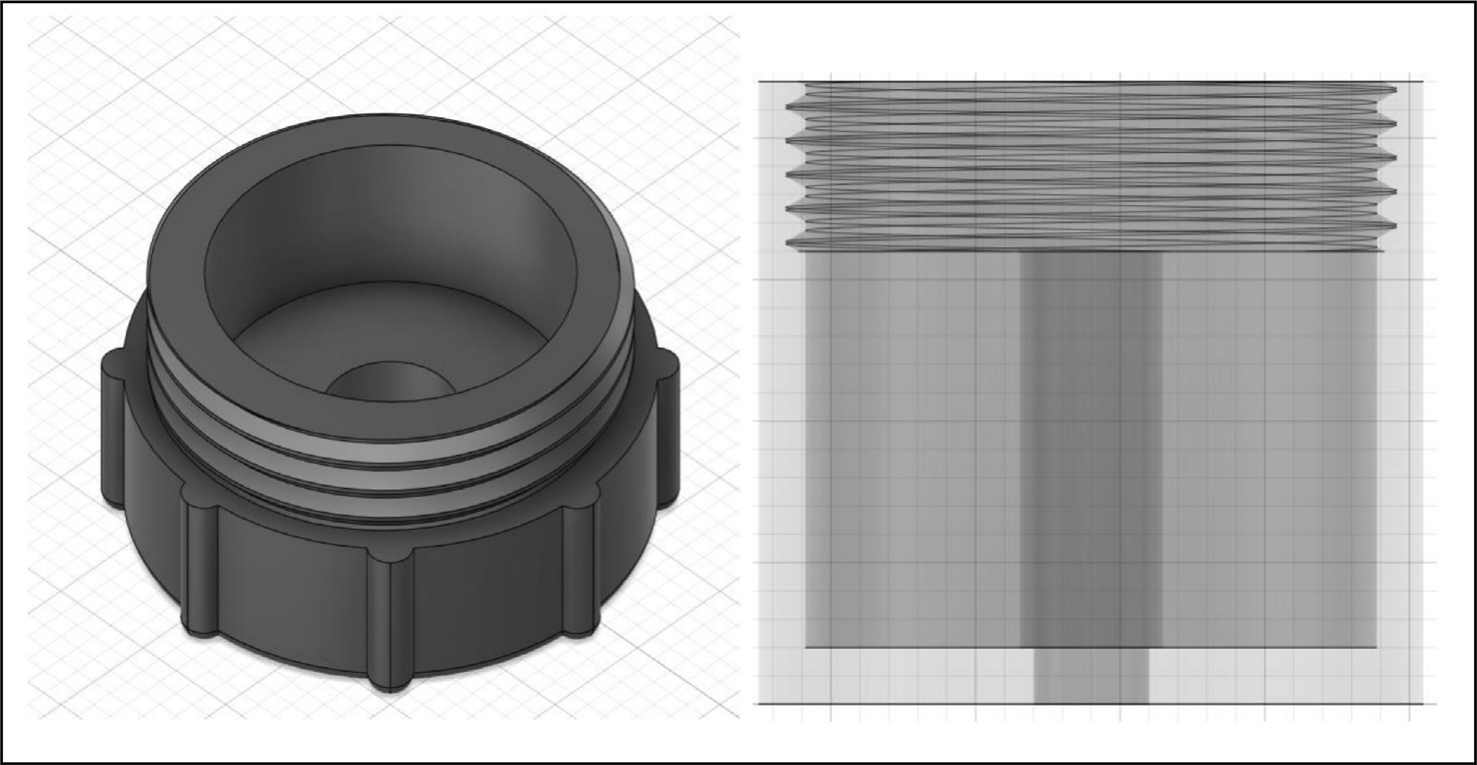
Water sampler.

The water sampler itself was designed to collect water samples from various depths and can be closed by a cap that can seal onto the top (See Fig. 3). The sampler can take up to 1000 ml (about 1 kg) volume of water, so that surplus water can be transported to laboratory facilities for further analysis, such as biochemical oxygen demand (BOD) which requires at least one day (BOD1) in typical BOD bottles having 60 ml or greater capacity (300 ml) as per Environmental Protection Agency (EPA) Method 405.1 and Standard Method [11]. Once water samples are collected by the airborne water sampler, in situ water quality measurement also took place for phosphate using a hatch kit [12]. Note that phosphates were recorded on the notebook while other water components (water temperature, pH, EC, and DO) are transmitted to the real-time cloud-based data sharing platform described later.

The proposed UASWQP could contribute UAS-based water quality studies in the sense that it can collect water samples at multiple points with multiple depths by navigating open waterbodies, ultimately benefiting the water research community in the following areas.

• Water quality monitoring and sampling activities to increase environmental stewardship at urban–rural interfaces
• Harmful Algae Bloom studies at lake and reservoir environment

## Design files

Design files are listed in Table 1. These supplementary files provide necessary information to produce the digital replicate of the sampling apparatus described in this article. For the 3D-printed version of the apparatus, the required .stl files can be found as Supplementary material of the present article. All files are detailed in the following table. These 3D models are easily replicable and scalable to be attached to different UAS platforms by adjusting parameters associated with architectural scale models in the respective 3D printers.

**Table 1:** Complete list of design supplementary files. Note that Open Source License follows CC BY 4.0.

Description	File type	File Name	Location of the file
M600 Male Adaptor	stl 3D file	IDX-SC-01	https://www.doi.org/10.17632/6nvcj4s2v4.1
Servo Compartment	stl 3D file	IDX-SC-02	https://www.doi.org/10.17632/6nvcj4s2v4.1
Spool	stl 3D file	IDX-SC-03	https://www.doi.org/10.17632/6nvcj4s2v4.1
Spool cap	stl 3D file	IDX-SC-04	https://www.doi.org/10.17632/6nvcj4s2v4.1
Sensor Box	stl 3D file	IDX-SB-01	https://www.doi.org/10.17632/6nvcj4s2v4.1
Raspberry Pi Plate	stl 3D file	IDX-SB-02	https://www.doi.org/10.17632/6nvcj4s2v4.1
Water sampler	stl 3D file	IDX-WS-01	https://www.doi.org/10.17632/6nvcj4s2v4.1
Sampler Cap	stl 3D file	IDX-WS-02	https://www.doi.org/10.17632/6nvcj4s2v4.1

## Bill of materials

The complete bill of materials (BOM) to replicate the UASWQP alongside the selection of sensors implemented is listed in Table 2. The listed components are not highly specialized and can be purchased from various local and online marketplaces, such as Amazon online stores.

**Table 2:** UASWQP Bill of Materials.

Designator	Component	Number	Cost per unit-currency	Total cost-currency	Source of materials	Material type
UASWQP	Unmanned Aircraft, DJI M600*	*1*	*$4,900*	*$4,900*	*DJI Store*	*Other*
	3D-printed Servo compartment	1	24.99 USD per kg	4.30 USD	Amazon	PLA filament, φ = 1.75 mm
	3D-printed Spool	1	24.99 USD per kg	1.8 USD	Amazon	PLA filament, φ = 1.75 mm
	3D-printed Spool Cap	1	24.99 USD per kg	0.2 USD	Amazon	PLA filament, φ = 1.75 mm
	Sensor Box	1	24.99 USD per kg	6.7 USD	Amazon	PLA filament, φ = 1.75 mm
	Raspberry Pi Plate	1	24.99 USD per kg	0.5 USD	Amazon	PLA filament, φ = 1.75 mm
	Water sampler	1	24.99 USD per kg	12.5 USD	Amazon	PLA filament, φ = 1.75 mm
	Sampler Cap	1	24.99 USD per kg	4.3 USD	Amazon	PLA filament, φ = 1.75 mm
	Raspberry Pi	1	64.4 USD	64.4 USD	Amazon	Other
	Sensor kits	1	500.0 USD	500.0 USD	Atlas Scientific	Other
	LTE IoT HAT	1	69.9 USD	69.9 USD	Sixfab	Other
	USB cables	1	6.9 USD	6.9 USD	Amazon	Other
	Runcam	1	66.0 USD	66.0 USD	Amazon	Other

## Build instructions

**Fig. 4:**
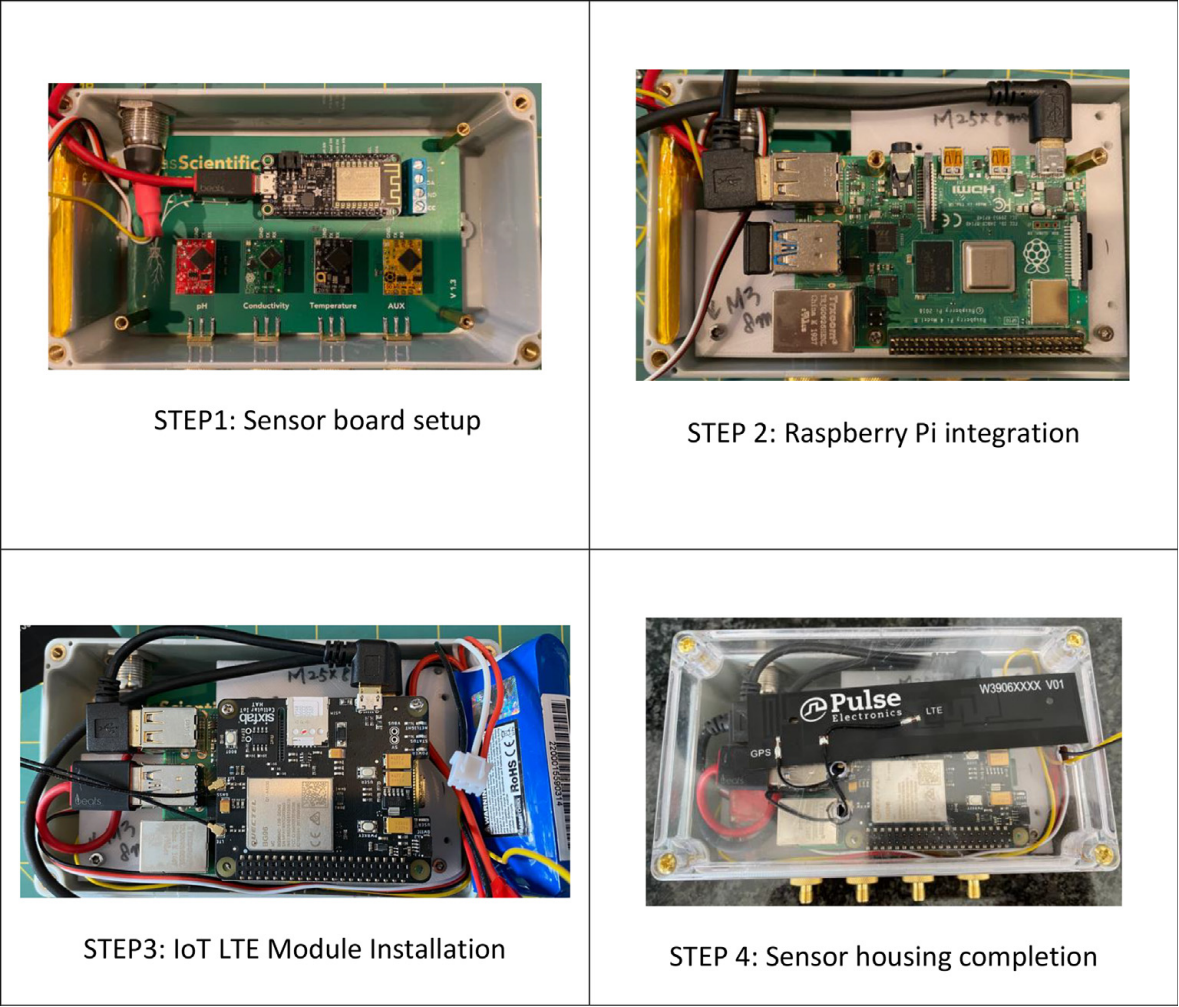
Sensor housing installation procedure.

The Wi-Fi sensing board (SB) built in Adafruit Huzzah32 (CPU), ESP32 communication module, and probes was used [13] to collect real-time water quality data, including water temperature, pH, and electronic conductivity (EC). Necessary efforts were made to calibrate the probes based on the manual before the probes getting in contact with water [13]. A Raspberry Pi 4 (RP) [14] was also used to transmit the collected data from SB in Arduino [15] to the cloud via Verizon LTE network [16]. Additionally, a cellular Internet of Thing (IoT) kit (LTE-M) [17] was then attached to the RP to minimize the form factor, fitting in the sensor housing as shown in Fig. 4. Unlike the servo, the Wi-Fi sensing board (SB) requires a separate battery to operate because it is hanging to M600 with wires to adjust height remotely during the flight. Thus, electrical wiring directly from M600 is not practical for the SB.

A separate two-cell (2S) LiPo battery was used to supply power voltage (5 V) for the Raspberry Pi (RP), while sufficient powers for Arduino Board was supplied from RP via a USB cable. Since the 2S LiPo battery supplies a nominal output voltage of 7.4 V which varies from 8.4 V when fully charged, down to 7.2 V, whereupon the internal battery regulator disables the battery from over discharge, a voltage regulator was used to provide 4 V with 3 Amps current for the target value. Based on the results from extended field testing, the 3 Amp h capacity of the battery is sufficient for this study for two hours without recharging or replacing with a fully charged battery.

## Operation instructions

Once the apparatus is attached to UASWQP securely, it can take off and fly to complete its mission. The pre-flight checklist (See Table 3) must be completed by the FAA certified remote pilot [18] prior to take off. Although there is no universal pre-flight checklist exists, Table 3 provides useful information for our specific mission for the water quality study using UASWQP. As soon as the pre-flight check is complete by Pilot-in-Command (PIC), the UASWQP takes off and conducts the flight mission. A low-cost video camera, such as a Runcam is attached to the M600 to send live streaming during the flight [19]. The built-in video transmitter and receiver in DJI Lightbridge allows Runcam to display field-of-view (FOV) on ground control station (GCS)’s screen (e.g., Apple iPhone/iPad or Android Phone/Tablet). Once the UASWQP approaches to the hotspot where monitoring and sampling take place, the PIC carefully operates and adjusts Kevlar taglines remotely to sink the sampler under water. The procedure of the UASWQP takes about 2–3 s to fill with water, while the sensors take an additional 10–30 s to properly collect water quality data while flying. Once the sampler of the UASWQP is filled up with water, the PIC can return it back to the takeoff location while the runcam provides a streaming video for the safe flight. A demo flight is presented at YouTube Video [20].

**Table 3:** The pre-flight checklist for UASWQP.

Category	Pre-flight^1^ Status
Weather Condition	Is it clear sky to guarantee line-of-sight (LOS) flight mission?
	Is wind speed (less than 8 m/s) adequate for safe flight?
Visual Inspection	Have you visually inspected whole system?
	Are all connection wires securely fastened?
	Are three key components (servo compartments, sensor box, sampler) properly positioned to avoid traffic hazard during take-off/landing?
	Are all three GPS in M600 upright positioned?
	Are six propellers spread well equally to reach the maximum thrust?
Ground Control Station (GCS)	Is the area clear and leveled for safe take-off and landing?
	Are there flight hazards, such as power lines, building, trees nearby?
	Is the area no drone zone?
	Is there unauthorized personnel nearby?
Operational Safety	Are all six batteries fully charged and secured?
	The separate battery for IoT sensors is fully charged and secured?
	Are all the switches in the transmitter in the default positions?
	Is the transmitter connected to ground control software, such as DJI Go on mobile phones (e.g., iPhone, Android)?
	Has the communication between the transmitter and M600 established?
	Is a safety communication protocol with the visual observer?

^1^The pre-flight checklist must be performed by Pilot-in-Command (PIC).

## Validation and characterization

### Demonstration site

**Fig. 5:**
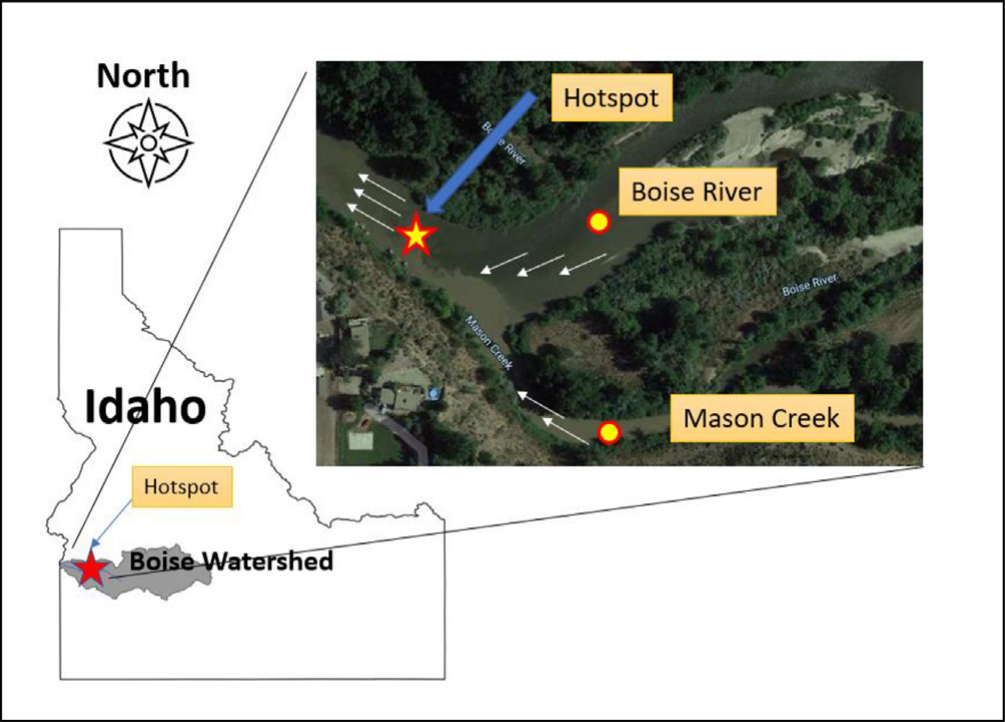
Demonstration Site in the Boise River, Idaho, USA.

A demonstration of the proposed UASWQP took place in the Boise River in the state of Idaho, USA. The Boise metropolitan area is the fastest growing cities in USA [21]. More than 40% of Idaho’s population lives in the greater Boise metropolitan areas, including Boise, Nampa, and Caldwell [22]. Since concerns of water quality degradation is an emerging issue associated with urbanization and agricultural activities nearby, a segment of the Boise River as shown in Fig. 5 is selected for the experimental site because the tributary agricultural return flow from Mason’s creek is contributing to water quality standards at the Boise River. The access to the site, however, is difficult due to physical constraints (e.g., steep slope, plant hazards). The site is situated in a low valley surrounded by forest and other foliage, and collecting water samples from the center of the Boise River, a hotspot and mixing zone, is challenging due to uneasy flow rates.

### Data visualization via cloud-based data sharing

**Fig. 6:**
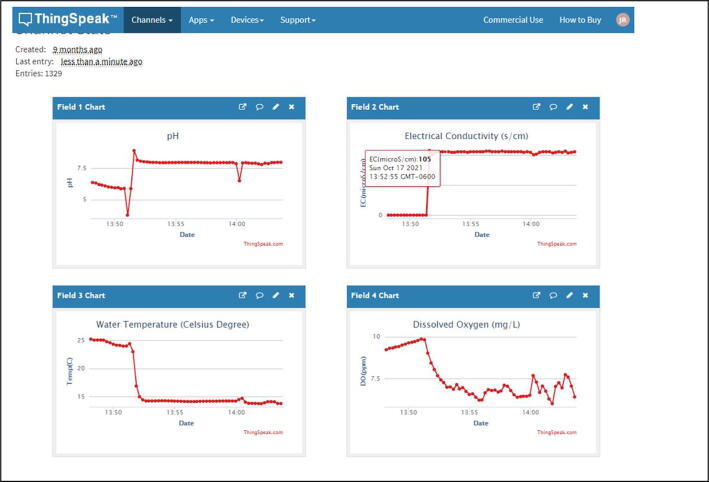
An example of ThingSpeak’s Graphical User Interface shows water quality data (water temperature, pH, EC, and DO).

**Fig. 7:**
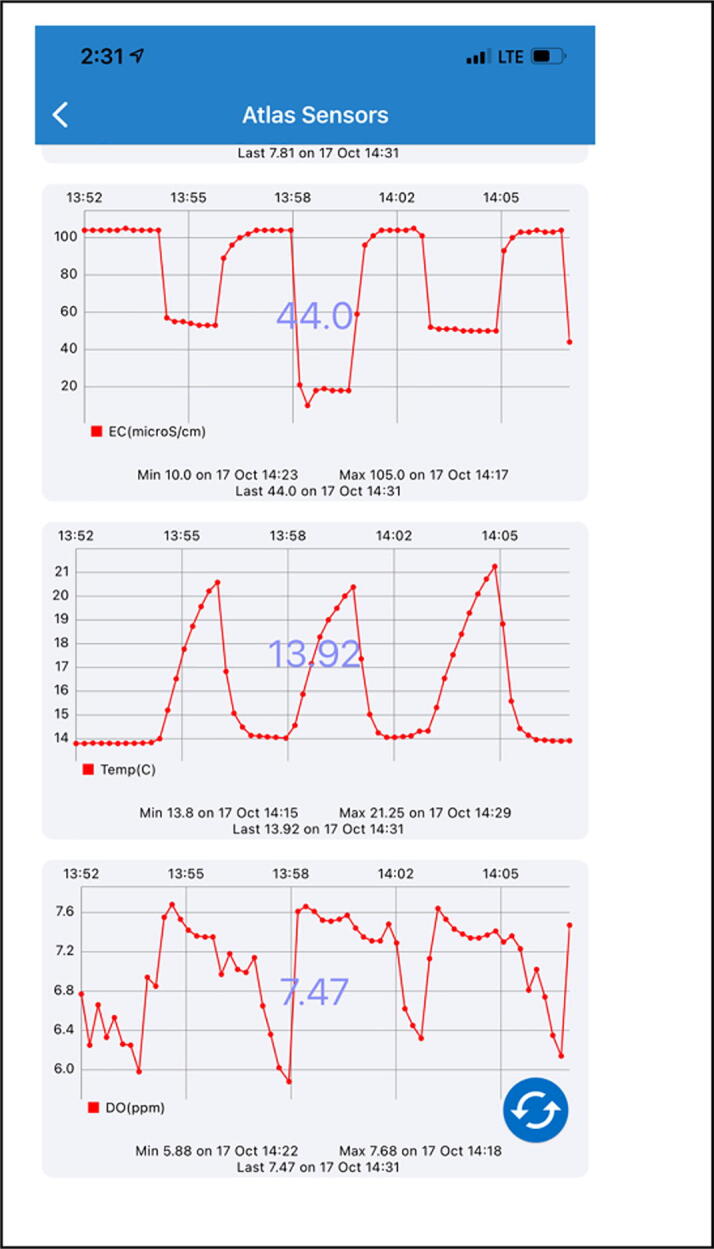
Screen capture from Mobile App in Thingview in iPhone.

ThingSpeak is used to send water quality data (water temperature, pH, and EC) to cloud networks for data sharing and visualization. ThingSpeak is an open-source IoT analytics platform service that allows to aggregate, visualize, and analyze real-time data streams in the cloud [23]. Thus, water quality data measured by IoT probes [13] are transferred to the flexible ThingSpeak cloud service [23] via Verizon LTE module [16] for data visualization. Fig. 6 shows an example of ThingSpeak’s graphical user interface (GUI) from a web browser’s interface (Web). The author has assigned four water quality components, including pH, Electrical Conductivity (EC), water temperature (Celsius) and dissolved oxygen (DO) for the first, second, third, and forth field, respectively. The graph in Fig. 6 shows the relationship between each water quality component and time, where changes are updated in 15 s intervals. Here, the average pH value is recorded at about 7.5 after first few minutes for travel flights and sensor stabilization. Likewise, through the web browser’s interface, additional visualization was achieved using mobile apps (e.g., ThingView in Apple iPhone) as shown in Fig. 7. Three water components EC, Water Temperature, and DO are displayed through the mobile app, while the pH can be visualized in the next screen (Not shown in this paper). Note that measurement time intervals of each water quality component can be noticeable between flights as shown in Fig. 7.

### Summary and additional thought

The application of UASWQP integrated with fast-moving technologies such as UAS, IoT, LTE wireless communication, and cloud web services is demonstrated to advance real-time water quality monitoring, sampling, and visualization activities at the environmental hotspot in the urban–rural interface. The off-the-shelf commercial UAS (DJI Matrice 600) was utilized to ensure that the UASWQP can sustain the necessary payload, including (1) the servo compartment, (2) sensor housing and probes, and (3) water sampler. The water sampler was designed to collect sufficient water samples up to 1000 ml (about 1 kg) for further analysis (e.g., BOD test) at laboratory facilities. The proposed UASWQP allows to send real-time water quality data, including water temperature, pH, and Electrical Conductivity (EC) to the ThingSpeak IoT Cloud web service via Verizon LTE wireless communication.

The proposed UASWQP platform can be used to collect field data at various waterways (rivers, lakes, reservoirs, and estuaries), especially where is inaccessible and/or hazard areas. To achieve fully autonomous mission flights, however, additional sensors, such as an optical sensor, collision avoidance sensor, and/or light detection and ranging (Lidar) sensor may be useful for better performances. Additionally, the development of a UAS swarm algorithm (group missions with multiple aircrafts) would be also useful for projects that need to generate heatmaps over large waterbodies. Coordination with other unmanned vehicles, such as unmanned surface vehicle (USV) or autonomous underwater vehicle (AUV) would be also potential opportunities to advance delineating geomorphology in reservoir and for aquatic habitat studies in agroecosystems. To improve visualization for larger projects, enterprise scale web services, such as Amazon Web Service or Microsoft Azure web apps could be considered to assure deliverables online seamlessly. Finally, the author anticipates that the proposed UASWQP could contribute UAS-based water quality studies near future in the sense that it has capability to collect water samples at multiple points with multiple depths autonomously by navigating open waterbodies, ultimately benefiting the water research community in years to come.

## Declaration of Competing Interest

The authors declare that they have no known competing financial interests or personal relationships that could have appeared to influence the work reported in this paper.
